# Cumulative and Persistently Elevated Serum Uric Acid-to-HDL Cholesterol Ratio and Incident Stroke Risk Among Middle-Aged Chinese Adults: A Landmark Analysis from CHARLS

**DOI:** 10.3390/jcm15218264

**Published:** 2026-10-23

**Authors:** Chongxia Zhong, Mingyue Wu, Kefan Mao, Yihai Liu, Biao Xu, Lina Kang

**Affiliations:** Department of Cardiology, Nanjing Drum Tower Hospital, Affiliated Hospital of Medical School, Nanjing University, No. 321 Zhongshan Road, Nanjing 210008, China

**Keywords:** UHR, cumulative exposure, repeated measurement, incident stroke, CHARLS

## Abstract

**Objective**: To examine the associations of baseline, cumulative average, and persistently elevated serum uric acid-to-high-density lipoprotein cholesterol ratio (UHR) with incident stroke among middle-aged Chinese adults. **Methods**: We used data from the China Health and Retirement Longitudinal Study (CHARLS). A dynamic-entry baseline cohort included 7113 stroke-free participants aged 45–60 years at cohort entry with available UHR and follow-up data. A repeated-UHR landmark cohort included 3438 participants with UHR measurements in both 2011 and 2015 who remained free of stroke through the 2015 assessment. Cox proportional hazards models were used to estimate hazard ratios (HRs) and 95% confidence intervals (CIs) for incident stroke. Baseline UHR was analyzed as a continuous variable and in quartiles. In the landmark cohort, cumulative average UHR and transition patterns across repeated measurements were assessed. **Results**: In the baseline UHR cohort, 477 incident strokes occurred during follow-up. After full adjustment, baseline UHR was associated with a higher risk of incident stroke (HR 1.082, 95% CI 1.013–1.155), and participants in the highest quartile had a higher risk than those in the lowest quartile (HR 1.458, 95% CI 1.115–1.905). In the repeated-UHR landmark cohort, 250 incident strokes occurred after the 2015 assessment. Cumulative average UHR was associated with incident stroke (HR 1.176, 95% CI 1.060–1.306). Compared with participants with persistently low UHR, those with persistently elevated UHR had a higher risk of incident stroke (HR 1.461, 95% CI 1.063–2.009). **Conclusions**: Among Chinese adults aged 45–60 years, higher baseline UHR, higher cumulative average UHR, and persistently elevated UHR were associated with a higher risk of incident stroke.

## 1. Introduction

Stroke remains a major cause of death and disability in China and imposes a substantial public health burden [1]. Many stroke risk factors, including hypertension, diabetes, dyslipidemia, smoking, obesity, and other cardiometabolic abnormalities, are potentially modifiable [2]. Because these risk factors often develop gradually during midlife, simple blood-based markers that reflect early vascular and metabolic risk may help identify individuals who could benefit from earlier prevention [3].

Serum uric acid and high-density lipoprotein cholesterol (HDL-C) are both closely related to vascular health. Higher serum uric acid has been associated with oxidative stress, endothelial dysfunction, inflammation, and a higher risk of stroke in cohort-based meta-analyses [4]. HDL-C participates in reverse cholesterol transport and has anti-inflammatory and antioxidative properties, although its association with stroke may differ by stroke subtype [5]. The serum uric acid-to-HDL-C ratio (UHR) has been proposed as a composite biomarker that may reflect both uric acid metabolism and lipid-related vascular risk [6].

Recent studies have reported associations between UHR and stroke, including cross-sectional analyses and single-baseline cohort studies [7,8]. However, most available evidence has relied on a single UHR measurement, which may not fully capture long-term uric acid-lipid imbalance. This limitation may be particularly relevant in midlife, when metabolic changes may accumulate before overt cerebrovascular events.

Previous CHARLS studies have examined baseline UHR in relation to incident stroke [9,10]. A distinct question is whether the average UHR across two assessments and the patterns of low and high UHR across those assessments are associated with subsequent stroke. Using CHARLS data, we examined baseline UHR and, in a 2015 landmark cohort, the average UHR and four low/high patterns across the 2011 and 2015 assessments in relation to subsequent stroke. In an additional exploratory analysis, we evaluated whether 2011 UHR provided incremental predictive information beyond 2015 UHR and clinical factors assessed in 2015.

## 2. Methods

### 2.1. Study Design and Population

The China Health and Retirement Longitudinal Study (CHARLS) is a nationally representative longitudinal survey of Chinese adults aged 45 years or older. The baseline survey was conducted in 2011 using a multistage probability sampling design across 150 county-level units and 450 village-level units, and recruited approximately 17,000 individuals from 10,000 households. Follow-up surveys were conducted in 2013, 2015, 2018, and 2020. The CHARLS study was approved by the Biomedical Ethics Review Committee of Peking University, and all participants provided written informed consent.

For the present analysis, we used a pooled dataset covering the 2011, 2013, 2015, 2018, and 2020 CHARLS survey waves. This dataset contained 25,873 unique participants identified by participant ID, of whom 17,708 had records in the 2011 survey. Because serum uric acid and HDL-C were available in the 2011 and 2015 waves, we constructed two analytic cohorts using these assessments. For the baseline UHR analysis, we used a dynamic-entry design in which each participant’s first eligible wave with available UHR data was defined as cohort entry. Participants were eligible if they were aged 45–60 years at cohort entry, were free of stroke at cohort entry, had complete covariate data, and had at least one subsequent follow-up assessment for incident stroke. Each participant contributed only once to the baseline UHR cohort. For participants with UHR available in multiple waves, the earliest eligible wave was used as cohort entry, and follow-up began after that assessment until incident stroke or the last completed follow-up.

**Figure 1:**
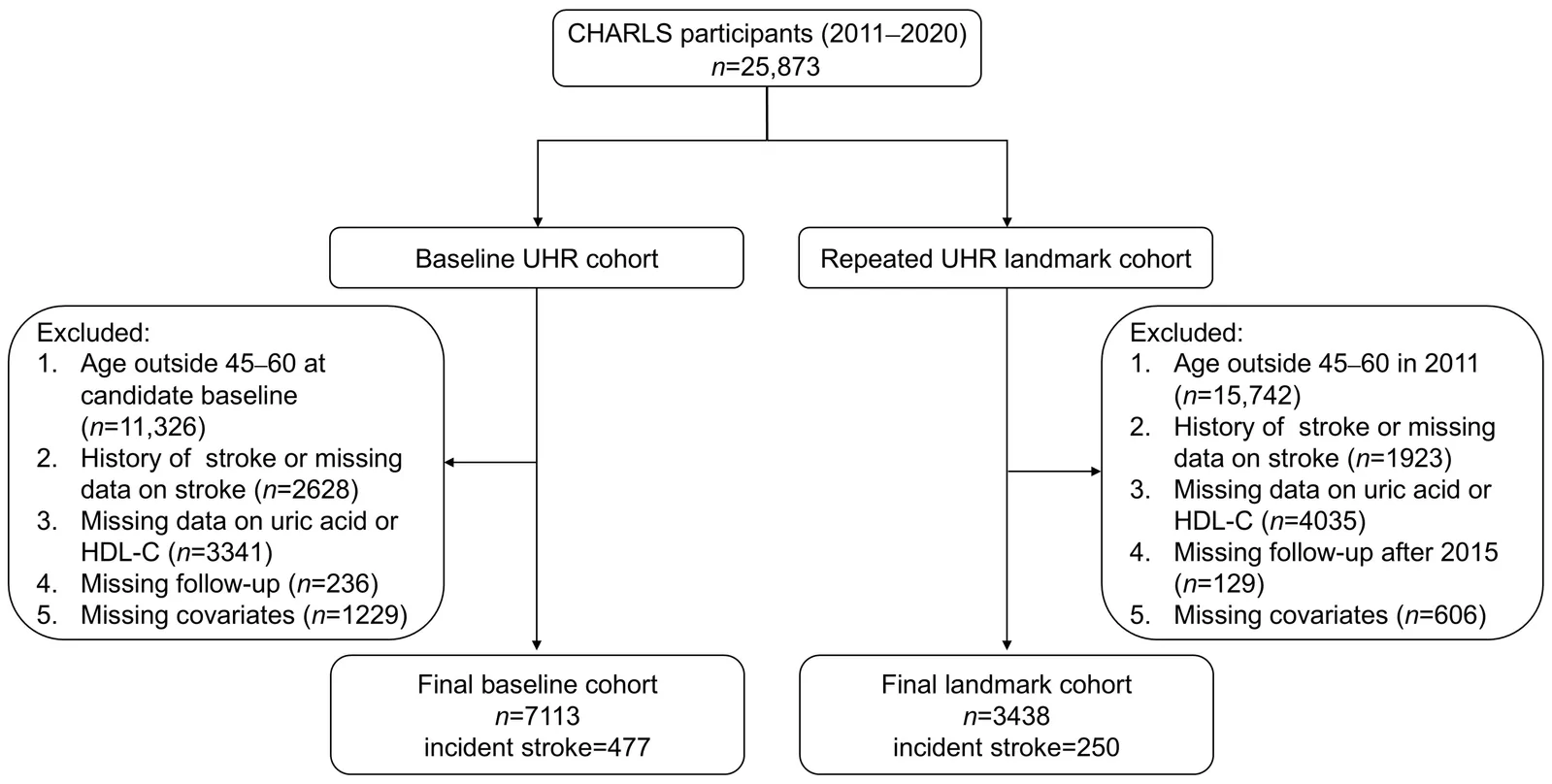
Study design and analytic cohorts.

For the repeated-measurement analysis, we constructed a landmark cohort using UHR measurements from 2011 and 2015. Participants were included if they were aged 45–60 years in 2011, remained free of stroke through the 2015 assessment, had UHR measurements in both 2011 and 2015, had complete covariate data, and had follow-up information after 2015. Follow-up for the landmark analysis started after the 2015 assessment. The detailed participant selection process is shown in Figure 1.

### 2.2. Assessment of Incident Stroke

The outcome was incident stroke during follow-up. At each CHARLS interview, participants were asked whether a physician had ever diagnosed them with stroke. For participants who reported stroke, event timing was assigned to the survey year of the first interview reporting stroke. In the baseline UHR cohort, participants with stroke at the cohort entry were excluded, and the first subsequent report of stroke was treated as an incident event. In the repeated-UHR landmark cohort, participants with stroke reported before or at the 2015 assessment were excluded; stroke events reported after the 2015 assessment were counted as incident events. Participants without a recorded stroke were censored at their last valid stroke assessment, including those subsequently reported deceased [11].

### 2.3. Assessment of Covariates

Trained interviewers collected baseline data through structured questionnaires, physical examinations, and laboratory tests, covering four domains: (1) demographic and lifestyle factors, including age, sex, marital status, rural residence, education level, smoking history, and drinking history; (2) body measurements, including body mass index (BMI), waist circumference, pulse, systolic blood pressure, and diastolic blood pressure; (3) medical history, including heart disease, hypertension, diabetes, dyslipidemia, kidney disease, liver disease, chronic lung disease, and cancer; (4) laboratory biomarkers, including serum uric acid, high-density lipoprotein cholesterol (HDL-C), low-density lipoprotein cholesterol (LDL-C), total cholesterol, triglycerides, glucose, glycated hemoglobin (HbA1c), creatinine, C-reactive protein, white blood cell count, platelet count, and hemoglobin. UHR was calculated as serum uric acid divided by HDL-C, with both expressed in mg/dL, and multiplied by 100. For the baseline UHR cohort, covariates were assessed at each participant’s cohort entry wave. For the main association analyses in the repeated-UHR landmark cohort, covariates were assessed at the 2011 baseline to preserve temporal ordering and avoid adjustment for potential intermediate changes between the 2011 and 2015 UHR assessments. For the incremental prediction analysis, the same clinical covariates were assessed at the 2015 wave.

Venous blood was collected by trained medical personnel. Participants were asked to fast overnight; non-fasting samples were also accepted, and fasting status was recorded. Plasma was separated, stored locally at −20 °C, and transferred for central storage at −80 °C before biochemical assays. In the 2011 wave, uric acid and HDL-C were measured at the Youanmen Center for Clinical Laboratory of Capital Medical University using the UA Plus method and an enzymatic colorimetric assay, respectively. In the 2015 wave, these biomarkers were measured at KingMed Diagnostics using uricase/catalase and direct methods, respectively. Both laboratories used daily quality-control specimens with weekly review of results [12]. Reported between-assay coefficients of variation for uric acid and HDL-C were 1.90% and 1.30% in 2011, and <2.32% and <3.14% in 2015, respectively. Laboratory procedures differed between waves; therefore, complete measurement equivalence cannot be assumed.

### 2.4. Statistical Analysis

Descriptive statistics were calculated for baseline characteristics. Continuous variables were expressed as medians with interquartile ranges [P25, P75], and categorical variables were presented as counts and percentages. Baseline characteristics across UHR quartiles were compared using the Kruskal–Wallis test for continuous variables and the chi-square test for categorical variables. Incidence rates were calculated as the number of incident stroke events per 1000 person-years.

Cox proportional hazards models were used to estimate hazard ratios (HRs) and 95% confidence intervals (CIs) for the associations of UHR with incident stroke. In the baseline UHR cohort, baseline UHR was analyzed both as a continuous variable per standard deviation (SD) increase and as quartiles, with the lowest quartile used as the reference group. A trend test across quartiles was performed by modeling the quartile variable as an ordinal variable. In the repeated-UHR landmark cohort, we assessed cumulative average UHR, calculated as the average of UHR measured in 2011 and 2015. Participants were classified into four UHR patterns (Low-Low, Low-High, High-Low, and High-High) using a cutoff of 8.830382, the median of the pooled 2011 and 2015 UHR measurements among the 3438 landmark participants. Values < 8.830382 and ≥8.830382 were classified as low and high, respectively. The pooled median was chosen to apply a common reference value across both assessments rather than wave-specific thresholds.

Three multivariable models were constructed: Model 1 was adjusted for age and sex. Model 2 was further adjusted for education level, marital status, rural residence, smoking history, and drinking history. Model 3 was additionally adjusted for BMI, hypertension, diabetes, heart disease, and kidney disease. Restricted cubic splines with three knots at the 10th, 50th, and 90th percentiles were used to assess the dose–response associations of baseline and cumulative average UHR with incident stroke, adjusting for Model 3 covariates. The median of each exposure was used as the reference. Overall associations and nonlinearity were assessed using likelihood ratio tests. Sensitivity analyses were performed in the repeated-UHR landmark cohort using sex-standardized UHR, winsorized UHR, log-transformed UHR, and sex-specific cutoffs for UHR transition patterns. Among landmark participants with complete 2015 covariate data, three Cox models included identical 2015 Model 3 covariates and continuous UHR measured in 2011, 2015, or both years. Using the same participants and follow-up, we compared the two-measurement model with each single-measurement model using likelihood ratio tests and Harrell’s C-index. C-index confidence intervals and paired differences were estimated using influence-function variances and normal approximation. Optimism-corrected C-indices were calculated using 500 participant-level bootstrap resamples (499 valid). Additional sensitivity analyses used the mean of wave-specific UHR z-scores, analyzed per SD increase, and UHR patterns defined by wave-specific median cutoffs (8.322678 in 2011 and 9.275000 in 2015; high UHR ≥ the corresponding cutoff), with the same landmark sample and covariate adjustment. All analyses were performed using R software (version 4.5.1), and two-sided *p* values < 0.05 were considered statistically significant. ChatGPT (OpenAI) was used to improve the clarity, grammar, and readability of the English text.

## 3. Results

### 3.1. Baseline Characteristics

A total of 7113 participants were included in the baseline UHR cohort (Figure 1). Of these participants, 5192 entered the cohort at the 2011 survey wave and 1921 at the 2015 survey wave. Overall, 3076 (43.2%) were men. The median age was 53.00 [49.00, 57.00] years, and the median UHR was 8.71 [6.61, 11.55]. Participants were categorized into quartiles of baseline UHR. Higher UHR quartiles included a larger proportion of men and showed higher BMI, waist circumference, systolic and diastolic blood pressure, serum uric acid, triglycerides, glucose, HbA1c, creatinine, C-reactive protein, white blood cell count, and hemoglobin, as well as lower HDL-C. The prevalence of hypertension, diabetes, dyslipidemia, ever smoking, and ever drinking also increased across UHR quartiles (Table 1). By the 2020 wave, 245 deaths were documented in the baseline cohort and 44 in the landmark cohort, including 23 and 9 participants, respectively, with stroke recorded in routine interviews. Among participants without a recorded stroke or documented death, 458 and 98, respectively, had incomplete final-wave stroke follow-up.

#### Dose-Response Associations

**Figure 2:**
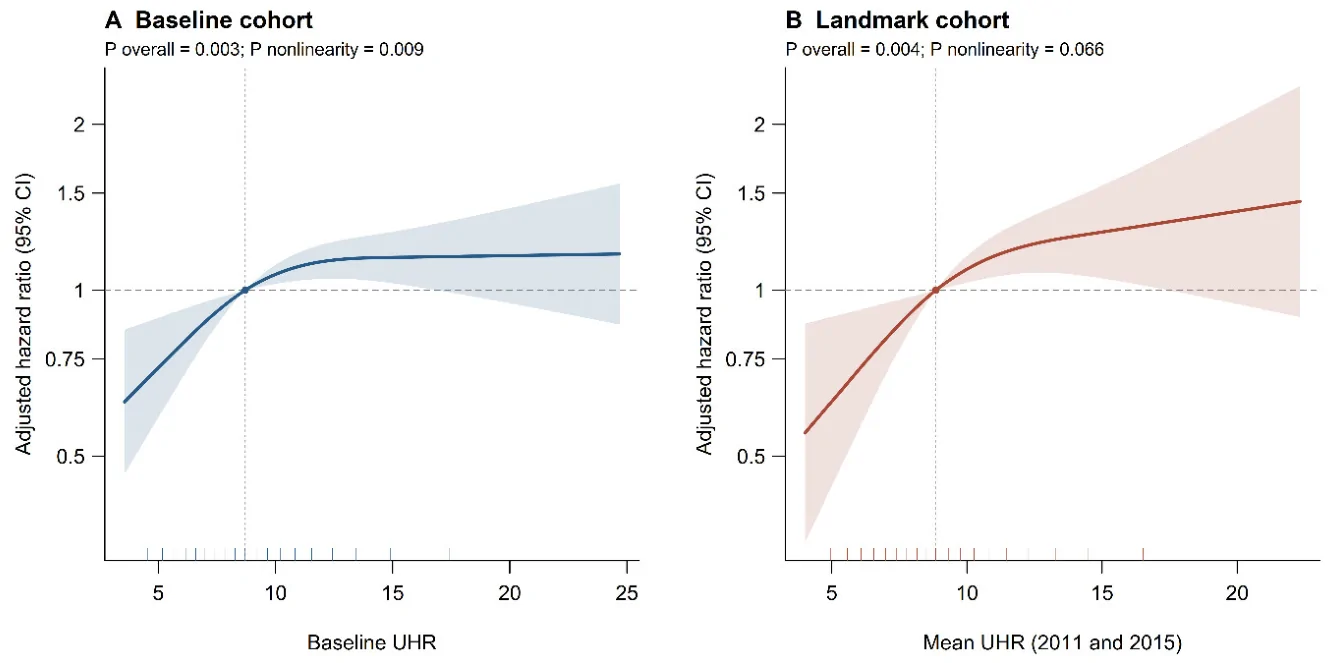
Dose–response associations of baseline and cumulative average UHR with incident stroke. Solid curves represent adjusted hazard ratios, and shaded areas indicate 95% confidence intervals. Horizontal dashed lines indicate HR = 1, and vertical dotted lines indicate the median UHR used as the reference in each cohort.

Restricted cubic spline analyses showed an association between baseline UHR and incident stroke, with evidence of nonlinearity (*p* for overall association = 0.003; *p* for nonlinearity = 0.009). The estimated association increased across lower-to-middle UHR values and flattened at higher values. Cumulative average UHR was also associated with incident stroke (*p* for overall association = 0.004), without statistically significant evidence of nonlinearity (*p* = 0.066) (Figure 2).

### 3.2. Association Between Baseline UHR and Incident Stroke

During a median follow-up of 9 [5,9] years, 477 incident strokes occurred in the baseline UHR cohort, corresponding to an incidence rate of 9.03 per 1000 person-years. In the fully adjusted model, each SD increase in baseline UHR was associated with a higher risk of incident stroke (HR 1.082, 95% CI 1.013–1.155, *p* = 0.018). Compared with participants in Q1, those in Q4 had a higher risk of incident stroke (HR 1.458, 95% CI 1.115–1.905, *p* = 0.006), with a significant trend across quartiles (*p* for trend < 0.001) (Table 2).

### 3.3. Repeated UHR Measurements and Incident Stroke

The repeated-UHR landmark cohort included 3438 participants with UHR measurements in both 2011 and 2015 who remained free of stroke through the 2015 assessment (Figure 1). Characteristics of participants included in and excluded from the landmark cohort are shown in Supplementary Table S2. During a median follow-up of 5 years after the 2015 assessment, 250 incident strokes occurred, corresponding to an incidence rate of 15.11 per 1000 person-years. Cumulative average UHR was calculated from the 2011 and 2015 measurements. Each SD increase in cumulative average UHR was associated with a higher risk of incident stroke after full adjustment (HR 1.176, 95% CI 1.060–1.306, *p* = 0.002).

Participants were categorized into four UHR transition patterns according to low or high UHR status in 2011 and 2015. Compared with the Low-Low group, the High-High group had a higher risk of incident stroke (HR 1.461, 95% CI 1.063–2.009, *p* = 0.020). The Low-High and High-Low groups had HRs above 1.0, but their confidence intervals included the null (Low-High: HR 1.349, 95% CI 0.927–1.964, *p* = 0.117; High-Low: HR 1.518, 95% CI 0.953–2.418, *p* = 0.079) (Table 3).

Among 3317 landmark participants with complete 2015 covariate data, 241 incident strokes occurred. Adding 2011 UHR to a model containing 2015 UHR and contemporaneous clinical factors did not significantly improve model fit (likelihood ratio *p* = 0.329). The apparent change in C-index was 0.00090 (95% CI −0.00211 to 0.00391), providing no clear evidence of improved discrimination (Supplementary Table S3).

### 3.4. Sensitivity Analyses

The repeated-UHR findings were consistent in sensitivity analyses. Cumulative average UHR remained associated with incident stroke when using sex-standardized UHR (HR 1.169, 95% CI 1.049–1.303, *p* = 0.005), UHR winsorized at the 1st and 99th percentiles (HR 1.227, 95% CI 1.083–1.390, *p* = 0.001), and log-transformation (HR 1.254, 95% CI 1.095–1.437, *p* = 0.001). The High-High pattern also remained associated with incident stroke when sex-specific cutoffs were used (HR 1.413, 95% CI 1.036–1.928, *p* = 0.029) (Supplementary Table S1). The wave-standardized mean UHR remained associated with subsequent stroke (HR per SD, 1.183; 95% CI, 1.063–1.316). Using wave-specific median cutoffs, the High-High group also had a higher hazard of stroke than the Low-Low group (HR, 1.544; 95% CI, 1.126–2.118) (Supplementary Table S4).

## 4. Discussion

In this prospective analysis of CHARLS participants aged 45–60 years at cohort entry, higher baseline UHR was associated with a higher risk of incident stroke. In the repeated-UHR landmark cohort, higher cumulative average UHR was also associated with subsequent stroke risk. Participants with persistently high UHR had a higher risk of incident stroke than those with persistently low UHR. These findings support an association between UHR levels assessed during midlife and subsequent stroke.

Our findings are consistent with recent studies linking UHR to stroke. Liang et al. examined 10,103 CHARLS participants and reported a higher incidence of stroke in the highest versus lowest UHR quartile after accounting for competing mortality (sHR 1.28, 95% CI 1.06–1.55) [9]. Li et al. also reported an association between baseline UHR and subsequent stroke in CHARLS and examined its joint association with BMI [10]. In a cross-sectional NHANES analysis, Su et al. found higher odds of prevalent stroke in the highest UHR quartile (OR 1.37, 95% CI 1.17–1.61) [13]. Their exploratory imaging-confirmed hemorrhagic stroke analysis showed a similar direction of association, although the adjusted association was not statistically significant. These studies support an association between UHR and stroke, but differ in population, outcome definition, and handling of competing mortality.

Our study adds a repeated-measurement perspective by examining the average of UHR values measured in 2011 and 2015 and patterns across these assessments among participants who remained stroke-free through 2015. This landmark design separates exposure assessment from subsequent stroke follow-up. The associations observed for cumulative average and persistently high UHR complement previous single-measurement findings, but do not establish superior predictive performance.

Previous studies have reported associations between UHR and stroke, but most available evidence has been based on cross-sectional data or single baseline UHR measurements [7]. A single UHR measurement may be affected by short-term biological variation and may not adequately represent long-term exposure. Our study extends prior findings by incorporating repeated UHR measurements and using a landmark design, in which UHR exposure was assessed before subsequent follow-up for incident stroke.

Compared with persistently low UHR, persistently high UHR was associated with a higher risk of incident stroke. Transient UHR patterns also showed point estimates above 1.0, but the estimates were imprecise and did not reach statistical significance. The association between cumulative average UHR and incident stroke was consistent across several sensitivity analyses, using sex-standardized UHR, winsorized UHR, and log-transformed UHR. In addition, the association for the High-High UHR pattern remained when sex-specific cutoffs were used, suggesting that the finding was unlikely to be solely explained by sex differences in UHR distribution.

UHR has been investigated as a composite marker related to inflammatory and metabolic disease. Previous studies have linked higher UHR with insulin resistance [14], metabolic syndrome [15], hypertension [16], arterial stiffness [17], and abdominal aortic aneurysm [18]. These findings suggest that UHR may reflect a broader adverse cardiometabolic and vascular profile rather than isolated changes in serum uric acid or HDL-C. Previous studies have suggested that uric acid may contribute to oxidative stress, inflammation, and endothelial dysfunction [19,20,21], whereas HDL-C may exert anti-inflammatory and antioxidative effects [22,23]. These findings provide possible biological explanations for the observed UHR–stroke association. However, these explanations remain hypothetical in the context of our study, as we did not directly test these mechanistic pathways. Our results establish epidemiological associations rather than mechanistic evidence.

The focus on adults aged 45–60 years may also be clinically relevant. Midlife is a period during which metabolic abnormalities, vascular dysfunction, and other modifiable risk factors may accumulate before overt cardiovascular or cerebrovascular events occur. Repeated assessment of UHR may help characterize individuals with sustained adverse metabolic profiles during this period. However, adding the 2011 UHR measurement to 2015 UHR and contemporaneous clinical factors did not demonstrate a clear improvement in discrimination. The repeated-measurement findings should therefore be interpreted as associations of two-time-point UHR profiles with subsequent stroke, rather than evidence supporting improved risk stratification.

This study has several limitations. First, stroke was self-reported without independent clinical validation or subtype information, and event timing reflected the first reporting interview rather than actual onset. Death dates were largely unavailable in the linked data. Censoring at the last valid stroke assessment may have missed strokes before death; outcome misclassification, informative censoring, and unaddressed competing mortality remain limitations. Second, because the repeated UHR analysis required serum uric acid and HDL-C measurements in both 2011 and 2015, the landmark cohort was smaller than the baseline cohort, and included participants differed from excluded participants in several characteristics. This may limit generalizability and introduce selection bias. Third, although UHR was measured repeatedly, only two biomarker assessments were available, which limited our ability to characterize detailed long-term UHR trajectories. The low/high classification was based on a sample-derived median rather than an established clinical threshold, which limits its generalizability and direct clinical applicability. Fourth, residual confounding cannot be excluded, although we adjusted for major demographic, lifestyle, and clinical factors. Fifth, UHR is a ratio-based marker and may be influenced by extreme values or low HDL-C levels; however, the results were consistent after winsorization and log-transformation. Finally, this analysis was conducted among middle-aged Chinese adults, and external validation in other populations is needed.

## 5. Conclusions

Overall, our findings suggest that cumulative and persistently elevated UHR during midlife were associated with a higher risk of incident stroke. These associations do not establish that repeated UHR measurements improve stroke risk prediction beyond a single contemporary measurement.

## Figures and Tables

**Table 1:** Baseline characteristics according to quartiles of baseline UHR among participants aged 45–60 years.

Characteristic	Overall	Q1 (*n* = 1779)	Q2 (*n* = 1778)	Q3 (*n* = 1778)	Q4 (*n* = 1778)	*p* Value
Age, years	53.00 [49.00, 57.00]	53.00 [48.00, 57.00]	53.00 [48.00, 57.00]	53.00 [49.00, 57.00]	53.00 [49.00, 57.00]	0.271
Male	3076 (43.2%)	427 (24.0%)	628 (35.3%)	849 (47.8%)	1172 (65.9%)	<0.001
Rural residence	4550 (64.0%)	1276 (71.7%)	1172 (65.9%)	1125 (63.3%)	977 (54.9%)	<0.001
Education level						<0.001
Illiterate	2725 (38.3%)	831 (46.7%)	720 (40.5%)	661 (37.2%)	513 (28.9%)	
Primary school	1477 (20.8%)	343 (19.3%)	379 (21.3%)	390 (21.9%)	365 (20.5%)	
Middle/high school	1914 (26.9%)	416 (23.4%)	451 (25.4%)	467 (26.3%)	580 (32.6%)	
College or above	997 (14.0%)	189 (10.6%)	228 (12.8%)	260 (14.6%)	320 (18.0%)	
Married or partnered	6706 (94.3%)	1649 (92.7%)	1682 (94.6%)	1663 (93.5%)	1712 (96.3%)	<0.001
Ever smoking	2615 (36.8%)	406 (22.8%)	561 (31.6%)	712 (40.0%)	936 (52.6%)	<0.001
Ever drinking	2808 (39.5%)	526 (29.6%)	628 (35.3%)	765 (43.0%)	889 (50.0%)	<0.001
Hypertension	2800 (39.4%)	571 (32.1%)	646 (36.3%)	711 (40.0%)	872 (49.0%)	<0.001
Diabetes	972 (13.7%)	179 (10.1%)	202 (11.4%)	245 (13.8%)	346 (19.5%)	<0.001
Heart disease	724 (10.2%)	151 (8.5%)	181 (10.2%)	180 (10.1%)	212 (11.9%)	0.009
Dyslipidemia	756 (10.8%)	107 (6.1%)	158 (9.0%)	196 (11.2%)	295 (16.9%)	<0.001
Kidney disease	435 (6.1%)	115 (6.5%)	104 (5.8%)	116 (6.5%)	100 (5.6%)	0.602
Liver disease	273 (3.8%)	59 (3.3%)	56 (3.2%)	79 (4.5%)	79 (4.5%)	0.069
Chronic lung disease	551 (7.8%)	126 (7.1%)	150 (8.5%)	137 (7.7%)	138 (7.8%)	0.512
Cancer	67 (0.9%)	11 (0.6%)	23 (1.3%)	18 (1.0%)	15 (0.8%)	0.200
BMI, kg/m^2^	23.74 [21.46, 26.35]	22.40 [20.55, 24.53]	23.50 [21.22, 25.81]	24.01 [21.61, 26.67]	25.29 [22.88, 27.77]	<0.001
Waist circumference, cm	85.00 [78.20, 92.30]	80.60 [74.80, 87.30]	84.00 [77.80, 90.00]	86.00 [79.00, 92.60]	90.50 [83.30, 97.20]	<0.001
Systolic blood pressure, mmHg	122.00 [111.00, 135.00]	118.50 [108.50, 131.50]	120.00 [109.50, 134.00]	122.50 [111.50, 135.00]	126.00 [115.50, 139.00]	<0.001
Diastolic blood pressure, mmHg	74.50 [67.00, 83.50]	72.50 [65.00, 81.00]	73.00 [66.00, 82.50]	75.50 [67.50, 83.50]	77.50 [70.50, 86.50]	<0.001
Pulse, beats/min	72.00 [65.50, 79.00]	71.00 [64.88, 78.00]	71.50 [65.00, 78.50]	72.50 [66.00, 79.00]	73.50 [67.00, 80.50]	<0.001
Serum uric acid, mg/dL	4.30 [3.58, 5.19]	3.30 [2.86, 3.76]	4.05 [3.57, 4.54]	4.63 [4.10, 5.20]	5.58 [4.88, 6.40]	<0.001
HDL-C, mg/dL	49.42 [41.31, 58.69]	61.78 [54.51, 71.13]	52.96 [47.17, 59.54]	46.33 [41.31, 52.12]	38.22 [32.86, 43.63]	<0.001
LDL-C, mg/dL	109.27 [88.80, 131.27]	110.04 [90.99, 132.99]	110.81 [91.62, 131.44]	111.58 [89.30, 132.99]	104.77 [82.63, 127.96]	<0.001
Total cholesterol, mg/dL	186.87 [165.08, 211.86]	190.98 [168.94, 215.34]	186.73 [165.46, 209.65]	184.41 [162.80, 211.86]	185.71 [162.37, 211.08]	<0.001
Triglycerides, mg/dL	109.74 [77.88, 165.49]	82.30 [62.83, 110.62]	99.12 [72.57, 136.29]	119.47 [86.73, 171.02]	169.03 [113.94, 266.39]	<0.001
Glucose, mg/dL	99.72 [91.80, 110.34]	97.92 [90.09, 107.46]	98.28 [90.09, 107.46]	99.90 [91.89, 110.16]	103.50 [93.78, 117.12]	<0.001
HbA1c, %	5.30 [4.90, 5.70]	5.20 [4.90, 5.50]	5.30 [4.90, 5.60]	5.30 [5.00, 5.70]	5.40 [5.00, 5.80]	<0.001
Creatinine, mg/dL	0.73 [0.64, 0.85]	0.67 [0.60, 0.75]	0.71 [0.63, 0.81]	0.76 [0.66, 0.87]	0.82 [0.71, 0.95]	<0.001
C-reactive protein, mg/L	1.00 [0.54, 2.08]	0.67 [0.40, 1.40]	0.88 [0.50, 1.77]	1.12 [0.60, 2.17]	1.51 [0.80, 2.90]	<0.001
White blood cell count, 10^9^/L	5.90 [4.90, 7.11]	5.60 [4.60, 6.80]	5.80 [4.80, 7.00]	5.90 [5.00, 7.10]	6.30 [5.33, 7.60]	<0.001
Platelet count, 10^9^/L	210.00 [166.00, 256.00]	213.00 [168.00, 259.00]	210.00 [167.00, 257.00]	211.00 [167.00, 256.00]	208.00 [162.00, 252.25]	0.116
Hemoglobin, g/dL	14.20 [12.90, 15.50]	13.70 [12.60, 14.90]	13.90 [12.80, 15.10]	14.20 [13.00, 15.50]	14.90 [13.70, 16.10]	<0.001
UHR	8.71 [6.61, 11.55]	5.46 [4.72, 6.05]	7.62 [7.11, 8.17]	9.94 [9.29, 10.66]	14.10 [12.70, 16.69]	<0.001

UHR: serum uric acid-to-HDL cholesterol ratio; HDL-C: high-density lipoprotein cholesterol; LDL-C: low-density lipoprotein cholesterol; BMI: body mass index.

**Table 2:** Association of baseline UHR with incident stroke among participants aged 45–60 years.

		Model 1		Model 2		Model 3	
Exposure	Cases/*N*	HR (95% CI)	*p* Value	HR (95% CI)	*p* Value	HR (95% CI)	*p* Value
UHR per SD increase	477/7113	1.136 (1.077–1.197)	<0.001	1.131 (1.073–1.192)	<0.001	1.082 (1.013–1.155)	0.018
UHR quartiles							
Q1	99/1779	Reference		Reference		Reference	
Q2	91/1778	1.012 (0.761–1.347)	0.933	1.008 (0.757–1.342)	0.959	0.943 (0.708–1.257)	0.691
Q3	127/1778	1.436 (1.100–1.874)	0.008	1.431 (1.095–1.869)	0.009	1.298 (0.993–1.696)	0.056
Q4	160/1778	1.820 (1.402–2.362)	<0.001	1.816 (1.395–2.364)	<0.001	1.458 (1.115–1.905)	0.006
*p* for trend			<0.001		<0.001		<0.001

Model 1 was adjusted for age and sex. Model 2 was further adjusted for education level, marital status, rural residence, smoking history, and drinking history. Model 3 was additionally adjusted for BMI, hypertension, diabetes, heart disease, and kidney disease.

**Table 3:** Association of cumulative average UHR and UHR transition patterns with incident stroke in the repeated-UHR landmark cohort.

		Model 1		Model 2		Model 3	
Exposure	Cases/*N*	HR (95% CI)	*p* Value	HR (95% CI)	*p* Value	HR (95% CI)	*p* Value
Cumulative average UHR per SD increase	250/3438	1.221 (1.116–1.335)	<0.001	1.232 (1.127–1.347)	<0.001	1.176 (1.060–1.306)	0.002
UHR transition pattern							
Low-Low	70/1282	Reference		Reference		Reference	
Low-High	46/613	1.413 (0.972–2.056)	0.070	1.413 (0.971–2.055)	0.071	1.349 (0.927–1.964)	0.117
High-Low	24/261	1.659 (1.043–2.639)	0.032	1.668 (1.049–2.655)	0.031	1.518 (0.953–2.418)	0.079
High-High	110/1282	1.649 (1.206–2.255)	0.002	1.686 (1.230–2.311)	0.001	1.461 (1.063–2.009)	0.02

Model 1 was adjusted for age and sex; Model 2 was further adjusted for education level, marital status, rural residence, smoking history, and drinking history; Model 3 was additionally adjusted for BMI, hypertension, diabetes, heart disease, and kidney disease. Cumulative average UHR was calculated as the average of UHR measured in 2011 and 2015. UHR patterns were defined using the pooled median of the 2011 and 2015 measurements (8.830382), with low UHR defined as <8.830382 and high UHR as ≥8.830382.

## Data Availability

The data used in this study were obtained from the China Health and Retirement Longitudinal Study (CHARLS). CHARLS is a nationally representative panel survey of Chinese adults aged 45 and older. The data are available at the official CHARLS website: https://charls.pku.edu.cn/.

## Supplementary Materials

The following supporting information can be downloaded at: https://www.mdpi.com/article/10.3390/jcm15218264/s1, Table S1: Sensitivity analyses for repeated-UHR measurements and incident stroke; Table S2: Baseline characteristics of participants included in and excluded from the repeated-UHR landmark cohort; Table S3: Incremental predictive value of repeated UHR measurements for incident stroke in the same landmark sample; Table S4: Sensitivity analyses using wave-standardized UHR and wave-specific median cutoffs in the landmark cohort.
