# Supplementary material for: Cumulative and Persistently Elevated Serum Uric Acid-to-HDL Cholesterol Ratio and Incident Stroke Risk Among Middle-Aged Chinese Adults: A Landmark Analysis from CHARLS
Source: J Clin Med. 2026 Oct 23;15(21):8264. doi: 10.3390/jcm15218264 (PMC13764184; doi:10.3390/jcm15218264)
Supplement: Supplementary file 1 [file jcm-15-08264-s001.zip › jcm-4538539-supplementary.pdf]

Supplementary Table S1. Sensitivity analyses for repeated-UHR measurements and incident stroke.

Sensitivity analysis	Contrast	HR (95% CI)	P value	Cases/N
Primary analysis	Cumulative average UHR per SD	1.176 (1.060-1.306)	0.002	250/3438
Sex-standardized cumulative average UHR	Per SD	1.169 (1.049-1.303)	0.005	250/3438
Winsorized cumulative average UHR	Per SD	1.227 (1.083-1.390)	0.001	250/3438
Log-transformed cumulative average UHR	Per SD	1.254 (1.095-1.437)	0.001	250/3438
Primary transition pattern	High-High vs Low-Low	1.461 (1.063-2.009)	0.020	250/3438
Sex-specific cutoff transition pattern	High-High vs Low-Low	1.413 (1.036-1.928)	0.029	250/3438

All estimates were derived from the repeated-UHR landmark cohort using fully adjusted Model 3. Winsorized UHR was truncated at the 1st and 99th percentiles; log-transformed UHR used the natural logarithm; sex-standardized UHR was calculated as sex-specific z scores. The sex-specific transition analysis used sex-specific pooled median cutoffs.

Supplementary Table S2. Baseline characteristics of participants included in and excluded from the repeated-UHR landmark cohort.

Characteristic	Included	Excluded	P value
Age, years	54.00 [49.00, 57.00]; n=3438	53.00 [48.00, 57.00]; n=4770	<0.001
Male	1400 (40.7%); n=3438	2437 (51.1%); n=4770	<0.001
Rural residence	2270 (66.0%); n=3438	2944 (61.7%); n=4770	<0.001
Education level			<0.001
Illiterate	1449 (42.1%); n=3438	1735 (36.4%); n=4762	
Primary school	694 (20.2%); n=3438	910 (19.1%); n=4762	
Middle/high school	874 (25.4%); n=3438	1347 (28.3%); n=4762	
College or above	421 (12.2%); n=3438	770 (16.2%); n=4762	
Married or partnered	3251 (94.6%); n=3438	4496 (94.3%); n=4770	0.587
Ever smoking	1177 (34.2%); n=3438	1976 (41.5%); n=4763	<0.001
Ever drinking	1253 (36.4%); n=3438	1954 (41.1%); n=4757	<0.001
Hypertension	1368 (39.8%); n=3438	1522 (32.1%); n=4736	<0.001
Diabetes	454 (13.2%); n=3438	415 (8.8%); n=4694	<0.001
Heart disease	331 (9.6%); n=3438	417 (8.8%); n=4732	0.221
Dyslipidemia	338 (10.0%); n=3390	405 (8.7%); n=4649	0.059
Kidney disease	181 (5.3%); n=3438	234 (5.0%); n=4725	0.560
Liver disease	110 (3.2%); n=3429	171 (3.6%); n=4717	0.338

Characteristic	Included	Excluded	P value
Chronic lung disease	241 (7.0%); n=3435	348 (7.3%); n=4746	0.615
Cancer	25 (0.7%); n=3435	39 (0.8%); n=4738	0.722
BMI, kg/m2	23.64 [21.42, 26.29]; n=3438	23.27 [21.09, 25.85]; n=3118	<0.001
Waist circumference, cm	85.00 [78.00, 92.00]; n=3434	84.00 [77.10, 91.00]; n=3160	0.001
Systolic blood pressure, mmHg	122.50 [111.00, 136.00]; n=3421	122.50 [112.00, 135.50]; n=3166	0.721
Diastolic blood pressure, mmHg	74.50 [67.00, 83.50]; n=3420	75.00 [67.50, 83.50]; n=3166	0.740
Pulse, beats/min	71.50 [65.00, 78.00]; n=3420	71.50 [65.00, 79.00]; n=3166	0.274
Serum uric acid, mg/dL	4.10 [3.43, 4.91]; n=3438	4.34 [3.60, 5.15]; n=2253	<0.001
HDL-C, mg/dL	48.71 [40.21, 58.76]; n=3438	49.10 [39.82, 59.54]; n=2254	0.662
LDL-C, mg/dL	112.89 [93.17, 135.31]; n=3427	112.89 [91.62, 135.70]; n=2252	0.244
Total cholesterol, mg/dL	190.59 [167.40, 214.56]; n=3435	188.27 [165.08, 212.73]; n=2252	0.063
Triglycerides, mg/dL	107.97 [76.11, 160.18]; n=3436	107.97 [77.00, 159.30]; n=2252	0.560
Glucose, mg/dL	101.88 [93.78, 111.60]; n=3433	101.88 [93.60, 112.68]; n=2243	0.675
HbA1c, %	5.10 [4.90, 5.40]; n=3423	5.10 [4.80, 5.40]; n=2305	0.074
Creatinine, mg/dL	0.72 [0.63, 0.84]; n=3431	0.76 [0.64, 0.88]; n=2243	<0.001
C-reactive protein, mg/L	0.90 [0.51, 1.86]; n=3438	0.91 [0.50, 1.94]; n=2253	0.473
White blood cell count, 10^9/L	5.90 [4.90, 7.20]; n=3383	6.00 [5.00, 7.30]; n=2263	0.146
Platelet count, 10^9/L	210.00 [165.00, 259.00]; n=3383	208.00 [166.00, 255.00]; n=2263	0.423
Hemoglobin, g/dL	14.20 [13.10, 15.50]; n=3384	14.50 [13.30, 15.90]; n=2262	<0.001
UHR in 2011	8.32 [6.32, 11.32]; n=3438	8.71 [6.54, 11.73]; n=2253	<0.001
UHR in 2015	9.28 [7.19, 11.91]; n=3438	9.52 [7.43, 12.19]; n=2141	0.001

Supplementary Table S3. Incremental predictive value of repeated UHR measurements for incident stroke in the same landmark sample.

Model	Cases/N	Apparent C-index (95% CI)	Optimism-corrected C-index
Clinical factors + 2011 UHR	241/3317	0.68655 (0.65356 – 0.71954)	0.66570
Clinical factors + 2015 UHR	241/3317	0.68673 (0.65413 – 0.71933)	0.66563
Clinical factors + 2011 and 2015 UHR	241/3317	0.68763 (0.65490 – 0.72036)	0.66563

Comparison	Added measurement	Δ C-index (95% CI)	Likelihood ratio P value
Both measurements vs. 2015 UHR alone	2011UHR	0.00090 (−0.00211 to 0.00391)	0.329
Both measurements vs. 2011 UHR alone	2015UHR	0.00108 (−0.00508 to 0.00724)	0.052

Supplementary Table S4. Sensitivity analyses using wave-standardized UHR and wave-specific median cutoffs in the landmark cohort.

Exposure	Cases/N	HR (95% CI)	P value
Wave-standardized mean UHR, per SD increase	250/3438	1.183 (1.063–1.316)	0.002
UHR patterns using wave-specific median cutoffs			
Low-Low	70/1298	Reference	
Low-High	29/421	1.234 (0.799–1.907)	0.343
High-Low	35/419	1.392 (0.925–2.094)	0.113
High-High	116/1300	1.544 (1.126–2.118)	0.007
